# Disparities in acute sepsis care: a systematic review

**DOI:** 10.1186/cc14102

**Published:** 2015-03-16

**Authors:** D Yamane, N Huancahuari, P Hou, J Schuur

**Affiliations:** 1 Brigham and Women's Hospital, Boston, MA, USA

## Introduction

Disparities in the incidence and outcomes of sepsis have been documented in observational studies but little is known about how these occur and how we might prevent them. Our objective is to identify disparities by race, language, gender, socioeconomic status, insurance status and geography in acute sepsis care in emergency department (ED) or ICU settings in the published literature.

## Methods

We performed a systematic review of disparities in sepsis care. The search strategy and inclusion and exclusion criteria were defined *a priori*. A medical librarian searched the entire MEDLINE (PubMed), EMBASE and Cinahl databases prior to 2013. One author reviewed all abstracts and a second author reviewed 10% of all abstracts for agreement. Both reviewers independently reviewed each included article using an explicit study review tool. We included studies that met the following inclusion criteria: ED or ICU setting; disparities due to race, language, gender, socioeconomic status, insurance status or geography; process of care measures (antibiotics, lactate, i.v. fluid resuscitation, central line placement, vasopressor use) or outcome measures (mortality, length of stay, complications, costs). We excluded studies involving organ-specific infectious conditions, pediatric populations, case reports, and review articles.

## Results

We identified 778 abstracts; yielding 31 for inclusion (*k *= 0.95), 26 of 31 studies were excluded due to quality issues. Five articles met our inclusion criteria. Only one of the studies [1] contained data on process of care measures, showing that central venous monitoring was less likely to occur in older patients. Three studies [2-4] showed that Black patients had a higher incidence of sepsis, a higher hospitalization rate, and higher mortality rate. Plurad and colleagues [5] reported that Asian patients had increased incidence of post-traumatic sepsis. Overall, Black patients with sepsis were younger, had lower socioeconomic status and were more likely to be cared for in urban settings compared with their cohorts.

## Conclusion

We found little published data addressing whether disparities due to race, language, gender, socioeconomic status, insurance status or geography exist in the acute care of sepsis. As sepsis is a leading cause of in-hospital mortality, future research should determine whether such disparities exist. Specifically, prospective studies of the process of care in sepsis management may further elucidate additional factors that may contribute to these disparities.
